# Supplementary figures and images for: Integrated post-genomic cell wall analysis reveals floating biofilm formation associated with high expression of flocculins in the pathogen Pichia kudriavzevii
Source: PLoS Pathog. 2023 May 17;19(5):e1011158. doi: 10.1371/journal.ppat.1011158 (PMC10228781; doi:10.1371/journal.ppat.1011158)

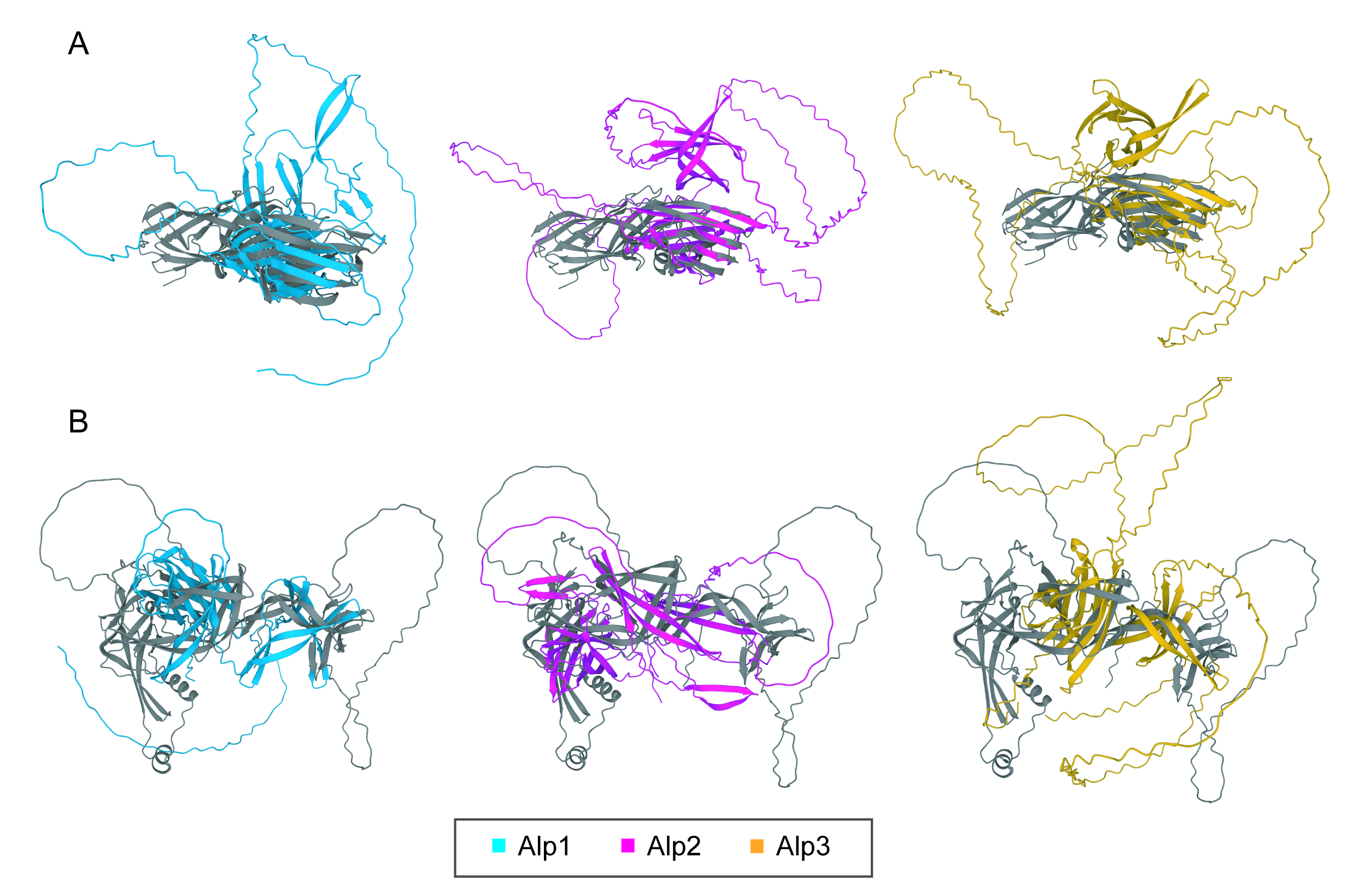

Supplement: S1 Fig — The C. albicans proteins are presented in grey color. (TIF) [file ppat.1011158.s001.tif]

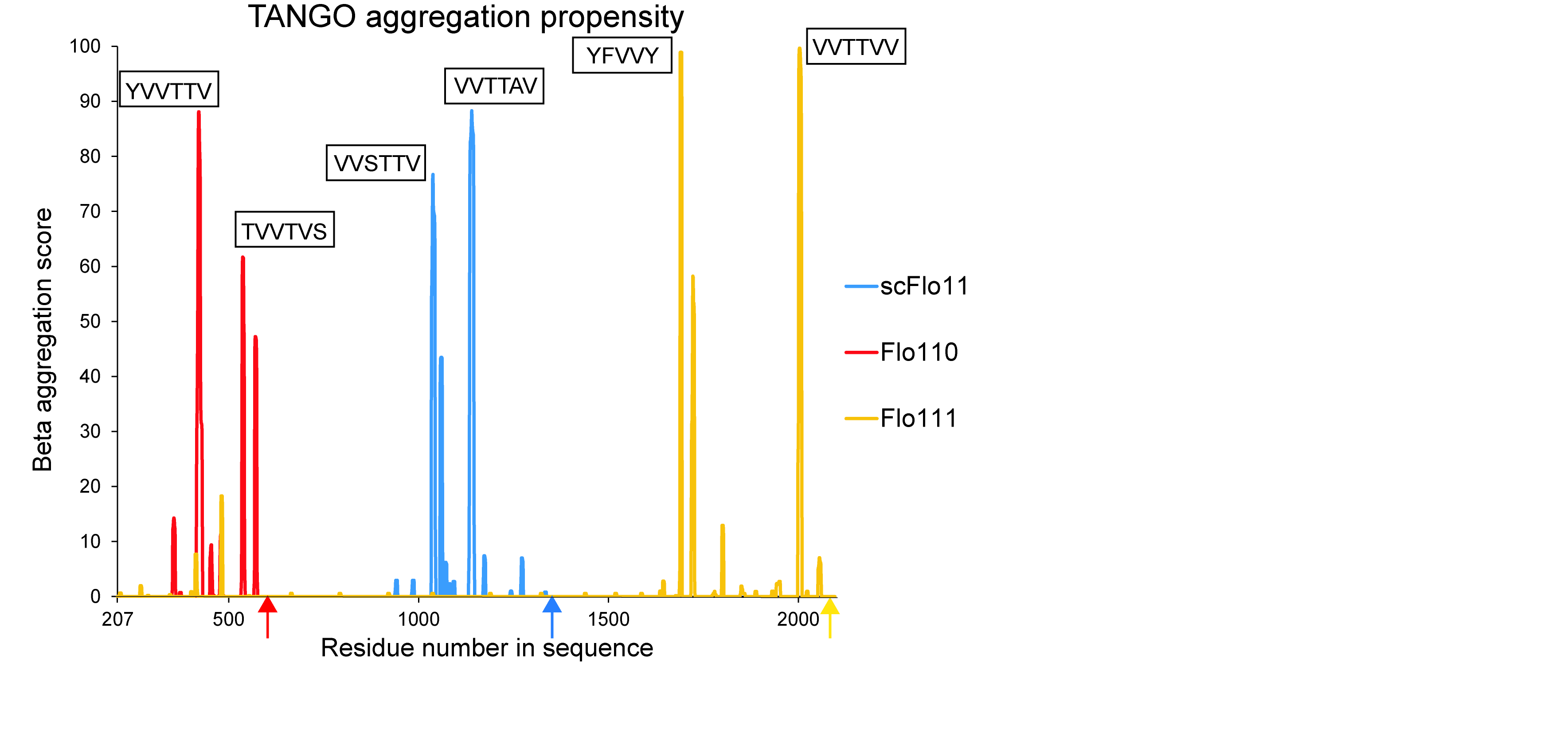

Supplement: S2 Fig — S. cerevisiae Flo11 is added for comparison. N-terminal domains of the proteins are eliminated for clarity. Vertical arrows indicate C-terminal ends of each protein. (TIF) [file ppat.1011158.s002.tif]
